# Dear Enemies Elicit Lower Androgen Responses to Territorial Challenges than Unfamiliar Intruders in a Cichlid Fish

**DOI:** 10.1371/journal.pone.0137705

**Published:** 2015-09-17

**Authors:** Rui F. Aires, Gonçalo A. Oliveira, Tânia F. Oliveira, Albert F. H. Ros, Rui F. Oliveira

**Affiliations:** 1 ISPA - Instituto Universitário, Lisbon, Portugal; 2 Instituto Gulbenkian de Ciência, Oeiras, Portugal; 3 Champalimaud Foundation, Lisbon, Portugal; University of Leicester, UNITED KINGDOM

## Abstract

In many territorial species androgen hormones are known to increase in response to territorial intrusions as a way to adjust the expression of androgen-dependent behaviour to social challenges. The dear enemy effect has also been described in territorial species and posits that resident individuals show a more aggressive response to intrusions by strangers than by other territorial neighbours. Therefore, we hypothesized that the dear enemy effect may also modulate the androgen response to a territorial intrusion. Here we tested this hypothesis in male cichlid fish (Mozambique tilapia, *Oreochromis mossambicus*) using a paradigm of four repeated territorial intrusions, either by the same neighbour or by four different unfamiliar intruders. Neighbour intruders elicited lower aggression and a weaker androgen response than strangers on the first intrusion of the experiment. With repeated intrusions, the agonistic behaviour of the resident males against familiar intruders was similar to that displayed towards strangers. By the fourth intrusion the androgen response was significantly reduced and there was no longer a difference between the responses to the two types of intruders. These results suggest that the dear enemy effect modulates the androgen response to territorial intrusions and that repeated intrusions lead to a habituation of the androgen response.

## Introduction

In territorial species, resident males have been shown to respond less aggressively towards an intrusion by a territorial neighbour than by a stranger male, a phenomenon known as the “dear enemy” effect [1,2]. From an evolutionary perspective this phenomenon can be seen as an adaptation for territorial males to adjust their behaviour according to the relative threat posed by the intruders.

The theoretical explanations for the dear enemy hypothesis rely on the familiarity existing between neighbours and on the relative threat posed by the different categories of intruders. The hypothesis based on the familiarity between neighbours proposes that territory owners are less aggressive towards neighbours either because familiarity decreases the risk of a role mistake (i.e. either contestant judging incorrectly its role as a likely winner/loser [3]) due to previous interactions among them (“role mistake hypothesis” [1]), or because they already have information on the resource holding power (RHP [3]) of their neighbours and therefore do not need further fights to get this information (“fighting to learn hypothesis” [4]). According to the latter explanation, the threat posed by stranger non-territorial floaters is higher than that posed by neighbours because the potential losses to strangers are higher (i.e. territory owners can lose both their territory and potential mates towards strangers but only potential males to neighbours that already have a territory) [2,4,5]. Moreover, assuming that territorial males hold information on the competitive ability of neighbouring males, obtained either actively from previous interactions or passively by eavesdropping on neighbours’ interaction with third parties [6], the level of uncertainty in the interactions with territorial neighbours is lower and thus they pose a lower challenge than stranger males. Therefore, the reduced aggressive response towards a “dear enemy” permits an economic territory defence without compromising its efficiency [7]. These hypotheses are not mutually exclusive and have been extensively tested across a wide range of taxa (e.g. crabs [8], fish [9], reptiles [10], birds [11]).

In terms of proximate mechanisms the “dear enemy” phenomenon requires the ability of the resident male to discriminate between familiar and unfamiliar intruders, together with a habituation response to the presence of neighbours, which would explain the lower response that they elicit [12,13]. Hormones may also play a role on the dear enemy effect by modulating the cognitive mechanisms mentioned above or by acting directly on the motivation of residents to engage in fights. Androgens have been shown to respond to social challenges in a wide range of species [14], and this response has been interpreted as a way to adjust the expression of androgen-dependent behaviours to social context [15,16]. For example, it has been shown that transient changes in androgen levels triggered by agonistic interactions influences competitive behaviour in subsequent interactions (e.g. winner effect [17,18]), that bystanders not directly involved in the interaction also respond hormonally to observed social interactions [19], and that environmental cues contingent with an interaction can trigger an anticipatory hormonal response in a Pavlovian fashion [20].

In this experiment, we test for the first time the hypothesis that the androgen responses to territorial intrusions may provide a simple mechanism underlying the dear enemy effect. Based on the evidence above, we predict a differential androgen response towards familiar vs. unfamiliar territorial intruders, so that territorial males should exhibit a lower androgen response when confronted with a familiar intruder, than when confronted with a stranger. Furthermore, we predict that due to habituation resident males should also gradually reduce their androgen response towards repeated territorial intrusions.

These predictions will be tested using an African cichlid, the Mozambique tilapia (*Oreochromis mossambicus*). In this species males establish territories in breeding aggregations to which they attract females to spawn with, and parental care is exclusively provided by the females [21,22]. Territorial males adopt a typical black velvet colouration [23] and build display sites (i.e. bowers) in the substrate that act as extended phenotypes used by females in mate choice [24]. Non-territorial males move around in breeding aggregations as floaters and either try to take over territories or to sneak fertilizations when females spawn with territorial males [25,26]. Previous work with other fish species (*Neolamprologus pulcher* [27]; *Cyprinodon variegates* [9]; *Cichlasoma nigrofasciatum* [7]) show that resident males are more aggressive towards unfamiliar males, however there is no information on how aggression towards neighbours and strangers varies over repeated territorial intrusions. This critical step to confirm the existence of a dear enemy effect will also be addressed in this experiment. Finally, we will also address a neglect potential confound in the test of the dear enemy effect, which is the modulation of the resident’s behaviour by variation in the intruders’ behaviour. Since neighbouring intruders are also more familiar with the resident male than stranger intruders, the former may act more boldly towards the resident and therefore induce higher levels of territorial defence, which do not reflect the mechanisms discussed above but rather a reflexive response to higher levels of aggression by the intruder.

## Material and Methods

### Animal Housing

Experimental fish (n = 15) were selected from a stock of individuals kept at the animal housing facilities of ISPA-IU. All fish were individually tagged with a magnetic transponder (Trovan ID 100: 2.2 x 11.5 mm; identification antenna: LID 500), which was implanted under anaesthesia (MS-222) in the peritoneal cavity.

Aquaria were equipped with a bottom filter and continuous aeration. A layer of sand of ca. 7 cm of height was deposited at the bottom of the aquaria, allowing males to dig spawning-pits that are essential for the full expression of their behavioural repertoire [28]. Water temperature was kept at 24 ± 2°C and the photoperiod regime was 12L: 12D. Fish were fed once per day with commercial fish flakes (Tropical Flake, Astra).

After the experiments all fish were returned to their original stock tank and none died or showed signs of chronic stress, during or after the experiment.

### Experimental Procedure

At the start of the experiment males were placed in individual tanks in which they could see one adjacent male and were allowed to become familiar with this neighbour over one week. After this period of time focal males received two 10 min. experimental intrusions on their territory per day, one from their neighbour and another from a stranger male. In order to study the “dear enemy effect” we monitored the agonistic behaviour of focal males during each intrusion test. As a non-invasive alternative to blood sampling we measured androgens from fish urine. Urine was only sampled after the intrusion tests of the 1^st^ and the 4^th^ (and last) day of the experimental protocol in order to minimize handling stress during the experiment. Androgen concentration in urine has been interpreted as integrating recent circulating androgen levels [29,30].

In total, 15 replicates were carried out. Each replicate consisted of: 1) a focal male who established a territory and remained in the same aquarium during the whole experiment; 2) males that were used as “intruders” in the territory of the resident, but kept their own territories in their home-tanks; two types of intruders were used: (a) one neighbour who established a territory in the same aquarium as the resident, with a transparent partition separating both males, and (b) four strangers: individuals that were housed in tanks in visual isolation from the focal male but otherwise in similar conditions to the neighbour.

In order to standardize motivational states between the two types of intruders, stranger males were kept in individual aquaria (50 x 40 x 30 cm) with visual access to each other by transparent partitions during a period of eight days prior to the start of the behavioural trials [29]. Similarly, residents and neighbours were placed in pairs in the test aquarium (100 x 40 x 50 cm) which had two divisions separated by a transparent sheet. In these aquaria, the resident male had more space than the neighbour (70 x 40 x 30 cm vs. 30 x 40 x 50 cm), so that the putative territory of strangers and neighbours was the same size. Thus residents and neighbours could interact visually and chemically with each other, while not having direct physical contact. Resident and neighbour males were allowed to habituate to the new aquaria also for a period of eight days. The experiments involved 10 min intrusions of either a neighbour or a stranger male at the territory of the resident. This duration was chosen because it allows the expression of the full repertoire of aggressive behaviour but it is too short for males to risk physical injury (RF Oliveira & AFH Ros, personal observations). Resident males received two intrusions per day, one in the morning and one in the afternoon with balanced order for intruder type. Before introducing an intruder in the focal fish tank, an opaque partition was placed against the transparent partition that separates the neighbour from the focal fish territories. At the end of the 10 min period, intruder males were caught and returned to their own aquarium. Focal males that were confronted with a neighbour in the first intrusion subsequently received a stranger intruder and vice versa. This set-up was repeated during the following three days but with alternating the order each day and with a balanced design [i.e. approximately half the residents received first a neighbour (n = 8) and the other half a stranger (n = 7)]. Both neighbours and all strangers were only used as intruders once per day.

In order to prevent the focal males from losing a fight and since body size is one of the best predictors of victory [31,32] we controlled the intruder’s size so that the resident would always be the largest male in each replicate. The body size of the 5 intruder males within each replicate was kept as similar as possible (Mean ± SEM for coefficients of variation across replicates = 3.0 ± 0.4%). Condition factor (K = body weight/(standard length)^3^) did not differ significantly between residents, strangers and neighbours [overall condition factor across the 3 groups (mean ± SEM) = 2.93 ± 0.06; Repeated measures ANOVA: F_(2,28)_ = 2.07, p = 0.15]. In addition, no difference in body length was found between males assigned as neighbours or strangers [t_(14)_ = .11, p = .92]. Together this data suggested that differences in behaviour or androgen levels elicited by the intrusion tests should not be due to variation in physical characteristics of the intruder males between groups.

### Behavioural measures

All experimental intrusion trials were recorded on video and subsequently analyzed using a multi-event recorder software (Observer XT, Noldus Inc., Holland). The video analysis was performed by an observer that was blind to the experimental treatments. The following behavioural categories were quantified based on the behavioural action patterns previously described for this species [23,25]: Approach—focal fish swims towards the intruder becoming closer than 1 body length; Displays—all occurrences of frontal displays (in a frontal position towards the opponent the fish erects the dorsal fin and opens the gill covers and the branchiostegal membrane) and lateral displays (in a parallel or antiparallel position towards the opponent the fish fully erects the dorsal and anal fins and fully spreads its caudal and pelvic fins; at its maximum intensity it can be combined with erecting the branchiostegal membrane, and with tail beating); Attack—all occurrences of chase, bite and carouseling (i.e. the two fish circle each other in an anti-parallel position often trying to bite each other); Fighting—all occurrences of mouthfighting (the opponents grip each others’ jaws, and having seized each other firmly by the mouth, they push and pull with tail beats) and pendelling (the two fish in a head to head position rush at each other with the dorsal and anal fins closed against the body; just before contact with the opponent the fish brakes to keep from colliding with it; often intersparsed with mouthfighting).

Frequency (number of occurrences per 10 min) and latency (time in seconds from the moment the intruder was introduced in the tank of the focal male until the queried behaviour was observed, with 10 min, i.e. trial duration, set as maximum latency) were registered for all the behavioural categories mentioned above. Duration (in seconds) was also measured for the behavioural categories that are states (i.e. displays and fighting).

### Urine sampling and analysis of androgen levels

Urine was collected by applying a small pressure on the lower part of the fish flanks behind the genital papilla [29]. Androgens were measured from fish urine collected within 5 minutes after the intrusion tests of the 1^st^ and the 4^th^ day of the experimental protocol, in order to minimize handling stress during the experiment Androgen concentration in the urine is interpreted as integrating recent circulating androgen levels [29,30]. We focused only on 11-ketotestosterone (KT) since it is the main androgen in teleost fish associated with the expression of male aggressive behaviour and of secondary sexual characters [33,34]. Urine samples were stored at -20°C until further processing. Free, glucuronated and sulphated fractions were extracted from each sample of 50 μl [29,35]. A radioimmunoassay (RIA) was used to measure the concentrations of KT in each of these fractions. The RIA characteristics, including the cross-reactivity of the antibodies used, have been reported before [36]. The intra and inter-assay variability was 8.2% and 11.6% respectively. Total levels of KT were calculated as the sum of all three fractions in each urine sample.

### Statistical analysis

All behavioural variables were logarithmically transformed [log_10_ (x+1)] to meet parametric test assumptions. In order to account for the influence of the intruder’s behaviour on the behaviour of the resident fish, an index [resident behaviour/(resident behaviour + intruder behaviour)] was calculated for all the paired resident male behavioural variables (i.e. attacks and displays). An escalation index was also calculated based the resident’s agonistic behaviour to territorial intrusions [attack frequency/(display frequency + attack frequency)].

To test the effects of the type of intruder on the resident behaviour, we have used a General Linear Model with type of intruder as a within-subjects factor (neighbour, stranger) and the behavioural variables as a repeated measures factor (4 levels: day 1, 2, 3, 4). A Linear Mixed Model with type of intruder (neighbour, stranger) and KT levels (day 1, day 4) as fixed factors, and the intercept as a random effect, was used to test the hormone response to the territorial intrusion, to avoid loss of data due to missing values. Planned comparisons were used within the statistical models to check for differences between strangers and neighbours in each day of the experiment (t-test for the General Linear Models, z-test for the Linear Mixed Model). The data used in the statistical analysis is available as supporting information (S1 Dataset).

### Ethics statement

Since the goal of this study was to study the effect of opponent familiarity in behavioural and hormonal responses to social challenges, and given the fact that the efficiency of the manipulation of familiarity cues in dummies or video-playbacks is questionable, and the response of this species to either of them is very limited (R.F. Oliveira, personal observation), we have used real intruder which elicited aggressive encounters. However, we have kept the sample size to a minimum and have limited the agonistic interactions to 10 min, following the “Guidelines for the treatment of animals in behavioural research and teaching” of the Association for the Study of Animal Behaviour (ASAB) [37]. No mortality of animals or serious physical injuries resulted from this experiment and all males were returned to their previous stock tanks after the experiments. All experimental procedures involved in this study were in compliance with the regulations on animal experimentation in Portugal and were approved by a permit (0421/000/000/2013) from the Portuguese Veterinary Authorities (Direcção Geral de Alimentação e Veterinária, Portugal).

## Results

### Effects of intruder familiarity and habituation on aggressive behaviour

**Fig 1:**
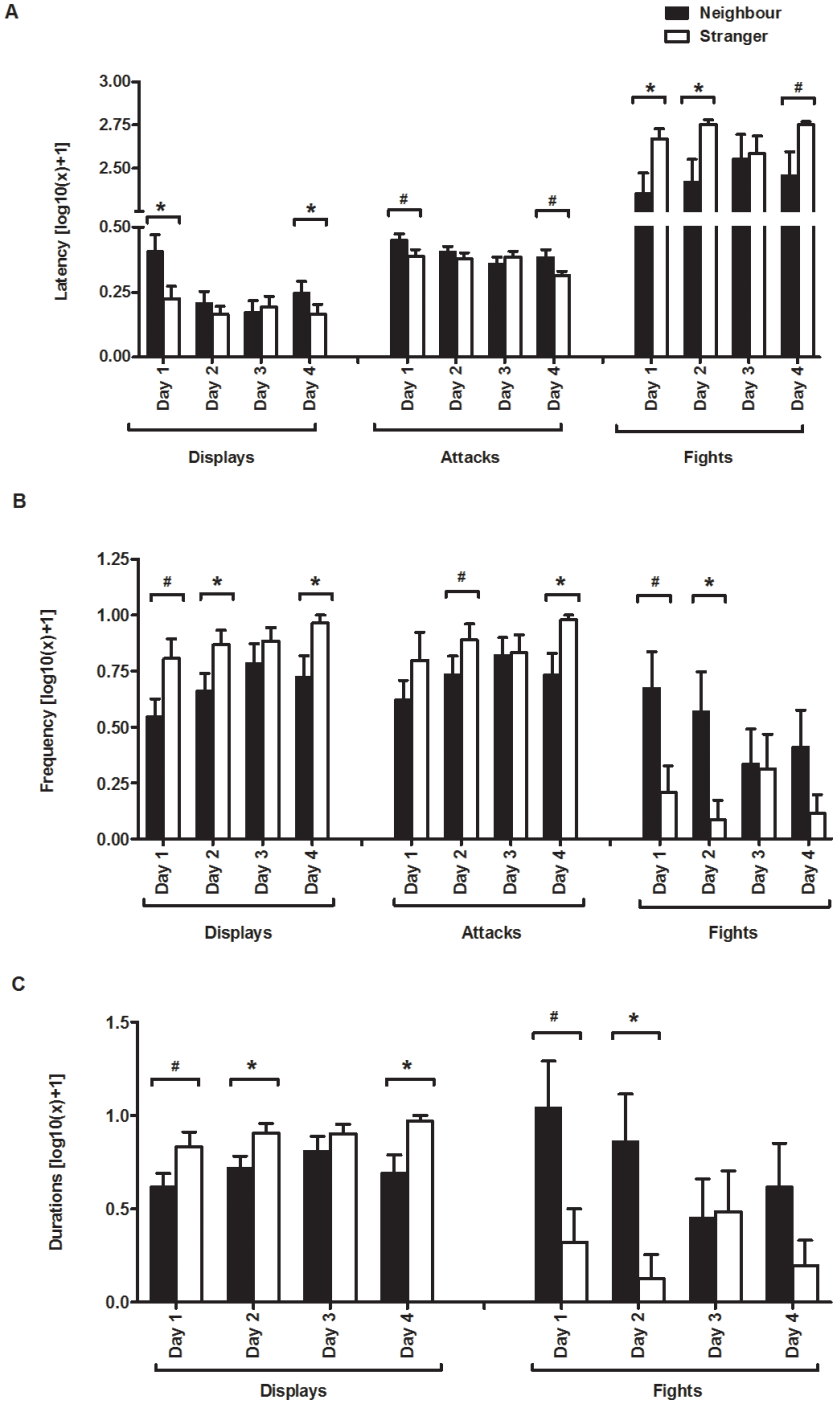
Aggressive behaviour displayed by resident males towards strangers and neighbours intruders during the 4 days of the experiment. A) Latency to displays, attacks and fights; B) Frequency for displays, attacks and fights; C) Duration of displays and fights; All plotted values for displays and attacks have been corrected for the influence of opponent’s behaviour in the interaction. *significant for p≤.05; # non-significant trend p≤.10.

Overall resident males displayed sooner [F_(1, 14) =_ 6.468, p = .023; Fig 1A], expressed more displays and attacks [Displays: F_(1, 14)_ = 10.053, p = .006; Attacks: F_(1, 14)_ = 5.046, p = .041; Fig 1B] and exhibited longer displays [F_(1, 14)_ = 11.239, p = .004; Fig 1C], towards stranger intruders than towards intruding neighbours. Both the latency to display and the latency to attack intruders decreased over the 4 days of the experiment [Displays: F_(3, 42)_ = 4.495, p = .007; Attacks: F_(3, 42)_ = 4.897, p = .005; Fig 1A], whereas display frequency increased with the course of the experiment [F_(3, 42)_ = 3.298, p = .029; Fig 1B]. A marginal non-significant trend for the frequency of attacks to increase over the 4 days of the experiment was also detected [F_(3, 42)_ = 2.248, p = .096; Fig 1B]. Shorter latencies to fight neighbours compared to strangers were detected on days 1 and 2, despite the lack of a significant main effect for this variable (F_(1, 14)_ = 4.079, p = .062; Fig 1A). Resident males also engaged more frequently in fights and these lasted longer when the intruder was a neighbour than when it was a stranger (Frequency: F_(1, 14)_ = 4.640, p = .049; Duration: F_(1, 14)_ = 4.869, p = .044; Fig 1B–1C). Planned comparisons to test differences in the behaviour of the intruder to each type of intruder on a daily basis, confirmed the main effects described above for some of the days, particularly days 1, 2 and 4, while no significant differences between the neighbour and stranger intrusions were detected on day 3 for any of the measures used in this experiment (Table 1).

**Table 1:** Statistical values for the differences in the resident males’ aggressive behaviours towards neighbour and stranger intruders over the course of the experiment

			Neighbour vs. Stranger						
		Day 1		Day 2		Day 3		Day 4	
	Measure	t	d	t	d	t	d	t	d
Displays	Latency	2.691*	.694	.797	.205	.455	.117	2.304*	.594
	Frequency	1.971#	.508	2.221*	.573	.883	.227	2.218*	.572
	Duration	1.858#	.479	2.523*	.651	.915	.236	2.578*	.665
Attacks	Latency	1.813#	.468	.865	.223	1.022	.263	2.019#	.521
	Frequency	1.107	.285	1.958#	.505	.089	.022	2.413*	.623
Fights	Latency	2.111*	.545	2.403*	.620	.168	.043	2.048#	.528
	Frequency	1.980#	.511	2.253*	.581	.108	.027	1.487	.383
	Duration	2.028#	.523	2.384*	.615	.113	.029	1.414	.365
Escalation	Index	2.324*	.600	.465	.120	.036	.009	.459	.118

t: values for contrasts (degrees of freedom = 14) between neighbour and stranger intrusions for each day of the experiment; d: effect size estimate (Cohen’s d).

*significant for p≤.05.

# non-significant trend p≤.10.

**Fig 2:**
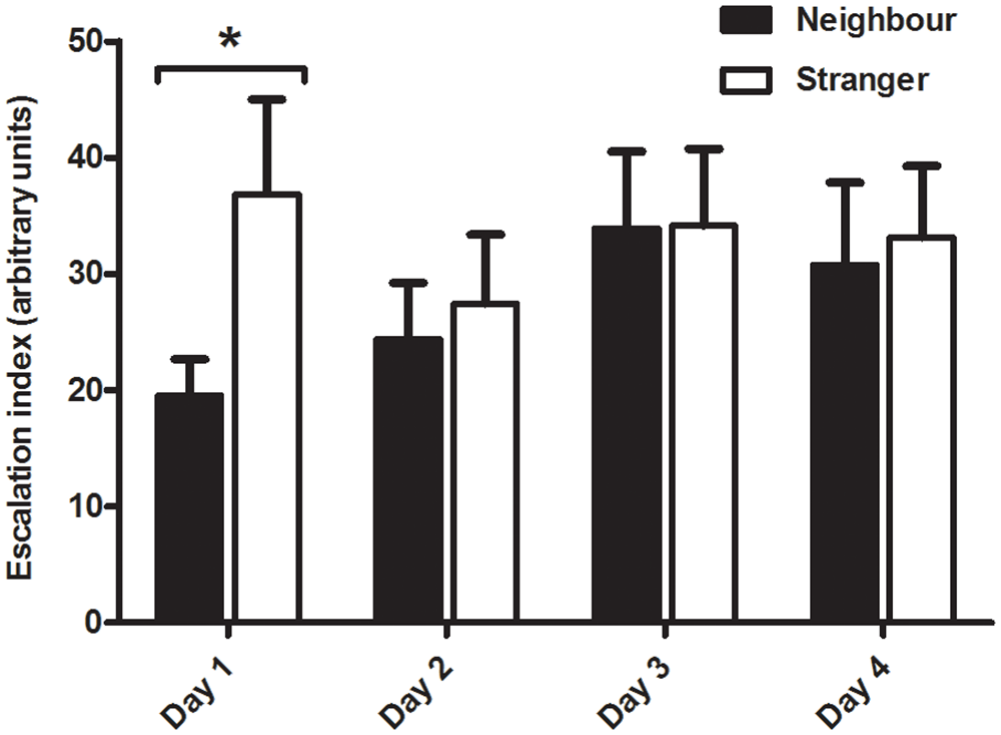
Resident males’ escalation index for intrusions by neighbours and strangers. *significant for p≤.05.

Although no main effect or interaction was detected for the escalation of fights, the resident males were more aggressive against stranger intruders than neighbours on day 1 (Intruder type: F_(1, 14)_ = 1.642, p = .220; Time: F_(3, 42)_ = ,81046, p = ,49525; Time x Intruder type: F_(3, 42)_ = 2.0978, p = .114; Table 1, Fig 2).

### Effects of intruder familiarity and habituation on KT levels

**Fig 3:**
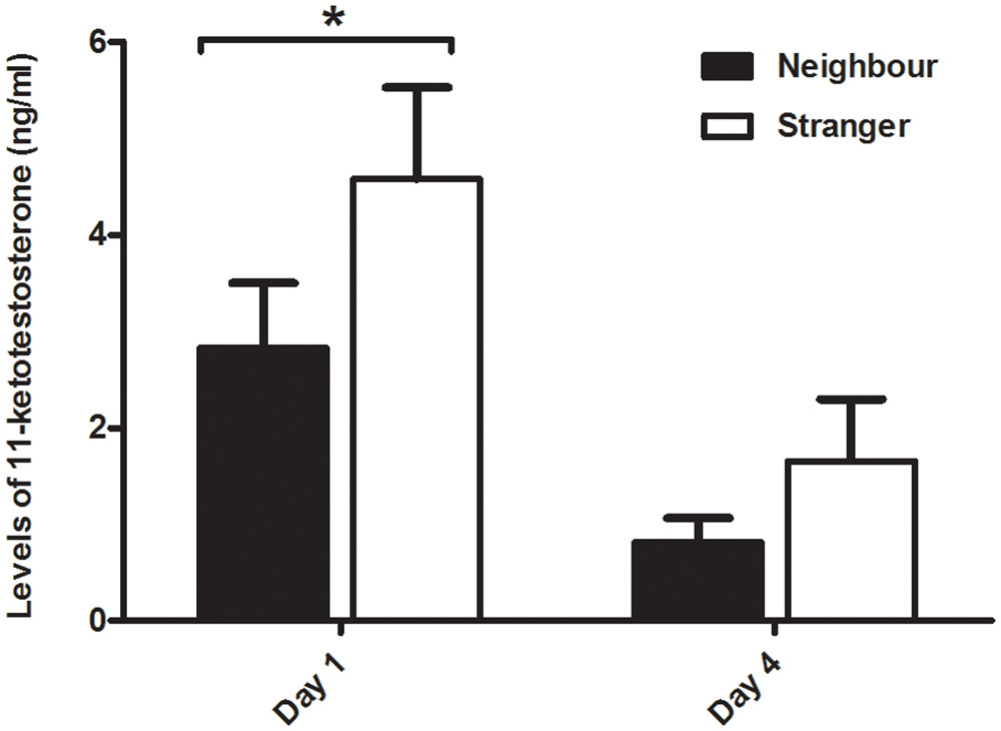
Resident males’ 11-ketotestosterone response to intrusions by strangers and neighbours on the first and last days of the experiment. *significant for p≤.05

Overall levels of KT lowered from day 1 to day 4 (F_(1, 29)_ = 15.219, p < .001). The resident male KT response to a territorial intrusion on day 1 was higher when the intruder was a stranger than when it was a neighbour (z = 1.928, p = .053, d = .674). This difference was no longer detected on day 4 (z = 1.034, p = .300, d = .494; Type of Intruder: F_(1, 29)_ = 3.853, p = .059; KT x Type of Intruder: F_(1, 29)_ = 0.508, p = .481; Fig 3).

## Discussion

In this experiment, we have tested the role of the androgen response to territorial intrusions as a mechanism underlying the dear enemy effect, using a paradigm of repeated territorial intrusions by neighbours and stranger males over the course of four days.

As predicted by the dear enemy hypothesis, resident males responded more aggressively towards territorial intrusions by strangers, as indicated by the differences in latency, frequency and duration for displays and attacks. Furthermore the escalation index confirms that the territorial intrusions by strangers elicited more aggression on the first day of the experiment. Unexpectedly, the fights against neighbours were longer and more frequent than against strangers. Since this experiment was carried out with real intruders and not with a standardized stimulus (e.g. dummies, video playbacks), it is possible that these paradoxical results for fights may be a consequence of differences in the behaviour of the two types of intruders (e.g. neighbour males being more familiar with the residents territory than strangers for whom it is novel). Moreover, all other behavioural measures were focused on the resident male and statistically corrected for the behaviour of the intruder, while no correction was possible for the fight measures, as it results from the behaviour of both males.

Contrary to the dear enemy predictions, we have not found evidence of a habituation effect on the residents’ behavioural response to repeated territorial intrusions by neighbours. Instead, the residents’ aggression on intrusions by neighbours approached those displayed towards strangers on day 3 of the experiment for all the measured parameters, suggesting that the repeated intrusions may have led to a shift in the strategy adopted by the resident males. We hypothesize, based on the threat assessment and the familiarity hypotheses for the dear enemy effect [2], that the repeated intrusions by neighbours caused a re-evaluation by the resident male of the threat posed by them to the resident’s male territory. This implies that although familiarity is an intervening component in the threat evaluation process, it is not a sufficient estimator [11]. The increased value of threat of the neighbours would hence explain the increase in aggressive behaviour by the resident male. This hypothesis is congruent with findings in other species, showing a dissipation of the dear enemy effect over the course of repeated territorial intrusions [38] or as a consequence of changes in the context in which the familiar intruder is presented to the resident male (e.g. after a recent intrusion [39]; territorial eviction [8]; presence of a female [9]; or seasonality [11]), suggesting that the dear enemy effect is not a fixed response, but a case of behavioural flexibility that can be modulated by the social environment.

In parallel to the dear enemy effect detected in the behavioural response, the resident males KT response to strangers was also higher than against familiar intruders on day 1. Furthermore, the KT response was lower at the end of the experiment, suggesting an habituation of the androgen response to repeated territorial intrusions. When compared to the behavioural findings, the results for KT match the findings for day 1, but are decoupled from those of day 4, in which most behaviours rebound in direction of a new dear enemy effect, after a period of similar aggression towards strangers and neighbours. These contrasting results confirm the previous finding that changes in social context (e.g. social instability [40]), may promote changes in the patterns of association between androgens and aggressive behaviour, which may become uncoupled.

Finally, it should be stressed that, in contrast to this experiment, in the natural environment resident males would also have access to information on the fighting ability of neighbours by eavesdropping on their agonistic interactions with third parties. This social phenomenon has been described for other fish species and effectively changes the fighting behaviour of bystanders [6], and thus may play a key role in the dear enemy phenomena. However, in the present study despite social eavesdropping not being accessible to resident males, a dear enemy effect was observed. This suggests that eavesdropped information on the relative fighting ability of the intruders, is not necessary for the dear enemy effect, and that threat assessment may rely on other social cues, such as familiarity and habituation/sensitization to intruders.

In summary, our results show for the first time that the dear enemy effect also modulates the androgen response to a social challenge, so that neighbours elicit a lower androgen response than strangers. Furthermore, this experiment along with other recent reports [8,38] suggests that the dear enemy effect is a flexible behavioural response modulated by social context and not a fixed response to familiar and unfamiliar intruders. These assumptions should be taken into account on future research in order to develop experimental designs that empirically test the predictions of the dear enemy effect in order to achieve a better understanding of this phenomenon.

## Supporting Information

S1 Dataset Behavioural and hormonal data for all individuals. (XLS)
